# The split apparent diffusion coefficient sign: A novel magnetic resonance imaging biomarker for cortical pathology with possible implications in autoimmune encephalitis

**DOI:** 10.1177/19714009231224416

**Published:** 2023-12-26

**Authors:** Riccardo Ludovichetti, Nathalie Nierobisch, Ngwe Rawlings Achangwa, Anthony De Vere-Tyndall, Jorn Fierstra, Regina Reimann, Claudio Togni, Robert Terziev, Marian Galovic, Zsolt Kulcsar, Nicolin Hainc

**Affiliations:** 1 Department of Neuroradiology, Clinical Neuroscience Center, 27243 University Hospital Zurich, University of Zurich, Switzerland; 2 Department of Neurosurgery, Clinical Neuroscience Center, 27243 University Hospital Zurich, University of Zurich, Switzerland; 3 Institute of Neuropathology, 27243 University Hospital of Zurich, University of Zurich, Switzerland; 4 Department of Neurology, Clinical Neuroscience Center, 27243 University Hospital Zurich, University of Zurich, Switzerland

**Keywords:** Autoimmune diseases, magnetic resonance imaging, diffusion magnetic resonance imaging, encephalitis, biomarkers

## Abstract

**Introduction:**

MRI is the imaging modality of choice for assessing patients with encephalopathy. In this context, we discuss a novel biomarker, the “split ADC sign,” where the cerebral cortex demonstrates restricted diffusion (high DWI signal and low ADC) and the underlying white matter demonstrates facilitated diffusion (high or low DWI signal and high ADC). We hypothesize that this sign can be used as a biomarker to suggest either acute encephalitis onset or to raise the possibility of an autoimmune etiology.

**Materials and Methods:**

A full-text radiological information system search of radiological reports was performed for all entities known to produce restricted diffusion in the cortex excluding stroke between January 2012 and June 2022. Initial MRI studies performed upon onset of clinical symptoms were screened for the split ADC sign.

**Results:**

25 subjects were encountered with a positive split ADC sign (15 female; median age = 57 years, range 18–82). Diagnosis included six herpes simplex encephalitis, three peri-ictal MRI changes, eight PRES, two MELAS, and six autoimmune (3 anti-GABA_A_R, two seronegative, and one anti-Ma2/Ta). Subjects were imaged at a mean 1.8 days after the onset of symptoms (range 0–8).

**Discussion:**

We present a novel visual MRI biomarker, the split ADC sign, and highlight its potential usefulness in subjects with encephalopathy to suggest acute disease onset or to raise the possibility of an autoimmune etiology when location-based criteria are applied. When positive, the sign was present on the initial MRI and can therefore be used to help focus further clinical and laboratory workup.

## Introduction

Encephalopathy is a broad term encompassing various types of brain disorders including those that are inflammatory, infectious, metabolic, or toxic in nature. Encephalopathic patients present a diagnostic challenge as symptoms are often wide-ranging and include altered mental status, focal neurological deficits, and seizures. MRI is the imaging modality of choice for assessing such patients with reported high sensitivities for many entities^1^; however, findings often lack specificity. Recognition of certain patterns or visual biomarkers can help focus further clinical and laboratory workup toward a certain category of disease.

One MRI pattern that can be seen in patients with encephalopathy is curvilinear areas of restricted diffusion involving the cerebral cortex. Diffusion weighted imaging (DWI) is an MRI technique from which the quantitative, mappable apparent diffusion coefficient (ADC) is derived. DWI/ADC characterize the degree of movement of water molecules and are most commonly used for assessing stroke. Multiple different pathomechanisms lead to a shift of relatively unrestricted water molecules from the interstitial space into the more constrained intracellular compartment resulting in cellular swelling; this can be characterized using DWI/ADC. Apart from stroke, restricted diffusion within the cortex has been described in infectious, metabolic, hemodynamic, and genetic diseases.^2^

Autoimmune encephalitis, once thought to be exceedingly rare, is now on par with that of infectious encephalitis in terms of incidence^3^ attributed to unprecedented antibody discovery rates and advances in laboratory diagnostics.^4^ However, diagnosis remains challenging,^5^ primarily due to medical professionals’ lack of awareness.^6,7^ Furthermore, clinical presentation can lead to misallocation of symptoms to, for example, psychiatric disease.^8^ A negative CSF or serum sampling result does not rule out autoimmune encephalitis and may necessitate repeat laboratory testing, further complicating the diagnostic process and delaying necessary treatment.^5^

In this context, we propose a new visual biomarker, namely the “split ADC” sign. With this sign, the cerebral cortex demonstrates restricted diffusion (high DWI signal and low ADC) and the underlying white matter demonstrates facilitated diffusion (high or low DWI signal and high ADC). We hypothesize that this sign can be used as a biomarker in encephalitis, indicating an acute onset or suggesting autoimmune encephalitis when location-based criteria are applied.

## Materials and methods

### Patient cohort

Ethical approval was obtained through the Institutional Review Board Kantonale Ethikkommission Zuerich, BASEC Nr. 2022-00041 prior to commencing the study. Informed consent was obtained for all patients included. A full-text radiological information system search of radiological reports was performed for all non-tumorous or tumor-like entities known to produce restricted diffusion in the cortex (stroke excluded) between January 2012 and June 2022 and included “seizure,” “status epilepticus,” “limbic,” “herpes,” “autoimmune,” “MELAS,” “PRES,” “Creutzfeldt Jakob,” “viral encephalitis,” “hypoglycemia,” “hypoxia/hypoxic,” and “hyperammonemia.” Abscesses were not considered. The initial MRI studies performed upon onset of clinical symptoms were screened for imaging abnormalities. Studies in which the above listed search terms were found only in the clinical information or query of the radiological report were not considered. All subjects included demonstrated restricted diffusion (low ADC) in the cortex and facilitated diffusion in the adjacent subcortical white matter (high ADC). Subjects with insufficient image quality were excluded. The diagnosis was confirmed for each patient via a review of the electronic patient record used to assess data including laboratory values, biopsy results, and clinical correlation.

### MRI findings

MRI of the brain was routinely performed with administration of intravenous contrast and consisted of axial T2-weighted FLAIR, T2W, DWI, SWI, and 3D T1W MPRAGE pre- and post-gadolinium, as per institutional protocol. Two neuroradiologists (RL and NH) with 4 and 8 years of neuroradiology reading experience assessed the images in consensus. The initial MRI performed upon patient presentation was assessed for the split ADC sign, beginning with identification of curvilinear cortical hyperintense signal change on DWI, correlated with low ADC compared to the surrounding unaffected cortex. Subcortical high ADC was identified in correlation with hyperintense signal change on FLAIR and T2-weighted sequences.

## Results

### Patient cohort

**Figure 1.**
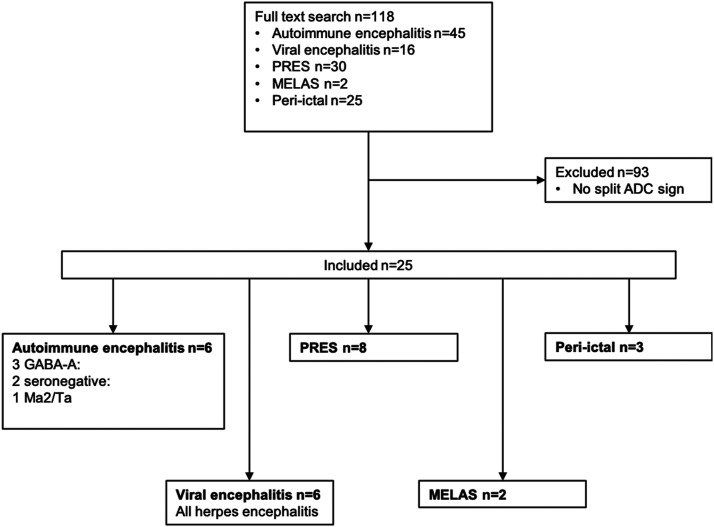
Flowchart of selection process. PRES = posterior reversible encephalopathy syndrome. MELAS = mitochondrial encephalomyopathy, lactic acidosis, and stroke-like episodes. ADC = Apparent diffusion coefficient. GABA_A_R = γ-aminobutyric acid receptor type A.

118 subjects were encountered upon full-text search, and of these, 25 subjects were included (Figure 1; 15 female; median age = 57 years, range 18–82). Diagnoses included six herpes simplex encephalitis, three peri-ictal MRI changes, eight PRES, two MELAS, and six autoimmune (3 anti-GABA_A_R, two seronegative, and one anti-Ma2/Ta). Signs and symptoms included generalized seizures (*n* = 8), impaired consciousness (*n* = 7), fever (*n* = 6), status epilepticus (*n* = 3), hypertension in pre-eclampsia (*n* = 2), focal neurological deficits (*n* = 2), focal seizure (*n* = 1), aphasia (*n* = 1), severe headache (*n* = 1), and visual disturbances (*n* = 1).

### MRI findings

**Figure 2.**
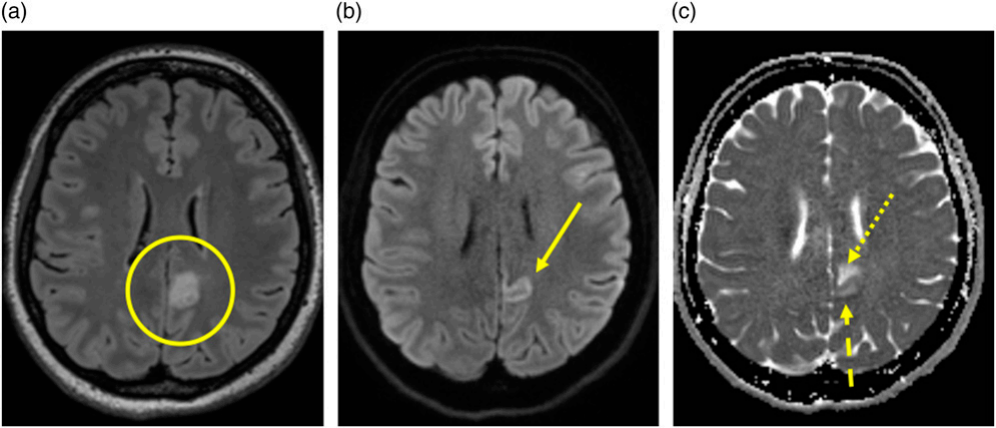
The split ADC sign in autoimmune encephalitis. (a) FLAIR image demonstrating signal change within the cortex and subcortical white matter of the left posterior cingulate gyrus (circle). (b) DWI image demonstrating signal change on DWI within the cortex (arrow). (c) ADC map demonstrating low ADC within the cortex (dashed arrow) and high ADC within the subcortical white matter (dotted arrow).

Subjects were imaged at a mean 1.8 days after the onset of symptoms (range 0–8) including 11 subjects being imaged on the same day of symptom onset. The split ADC sign (Figure 2) is characterized by low ADC (restricted diffusion) in the cortex and high ADC within the subcortical white matter. The location of the split ADC sign for all subjects is described in Table 1.

**Table 1.** Location of the split ADC sign for all entities included.

Entity	Location summary
Autoimmune (anti-GABA_A_R)	• 1 scattered cingulate gyrus bilaterally (bilateral anterior and left posterior), lateral temporal lobe bilaterally (asymmetric).
	• 1 unilateral scattered frontal, temporal, and insular.
	• 1 bilateral symmetric temporoparietal
Autoimmune (seronegative)	• 1 scattered frontal, parietal, and temporal bilaterally
	• 1 left frontal and right anterior cingulate gyrus
Autoimmune (Ma2/Ta)	• Unilateral temporal lobe
Viral (herpes simplex encephalitis)	• 3 unilateral temporoinsular
	• 2 bilateral asymmetric temporopolar
	• 1 unilateral temporoinsular and insular
PRES	• 5 bilateral symmetric parieto-occipital
	• 2 bilateral asymmetric parieto-occipital
	• 1 bilateral symmetric frontal and parieto-occipital
MELAS	• 1 unilateral lateral temporal
	• 1 bilateral asymmetric frontal
Peri-ictal	• 1 right hemisphere and left cingulate gyrus with bilateral pulvinar and hippocampus involvement
	• 1 left cingulate gyrus and ipsilateral pulvinar and hippocampus involvement
	• 1 unilateral temporooccipital

### Biopsy results

In a subject with proven anti-GABA_A_R receptor encephalitis, biopsy of the right frontal cortex revealed increased expression of glial fibrillary acidic protein (GFAP) signifying astroglial activation and gliosis. CD45+lymphocytes were identified in the perivascular space and within the brain parenchyma. CD68+ macrophages with microglial nodules were identified. Olig2 staining revealed perineuronal satellites without evidence of viral particles. Furthermore, CD3+, CD4+, and CD8+ lymphocytes were identified. No CD20+ lymphocytes.

**Figure 3.**
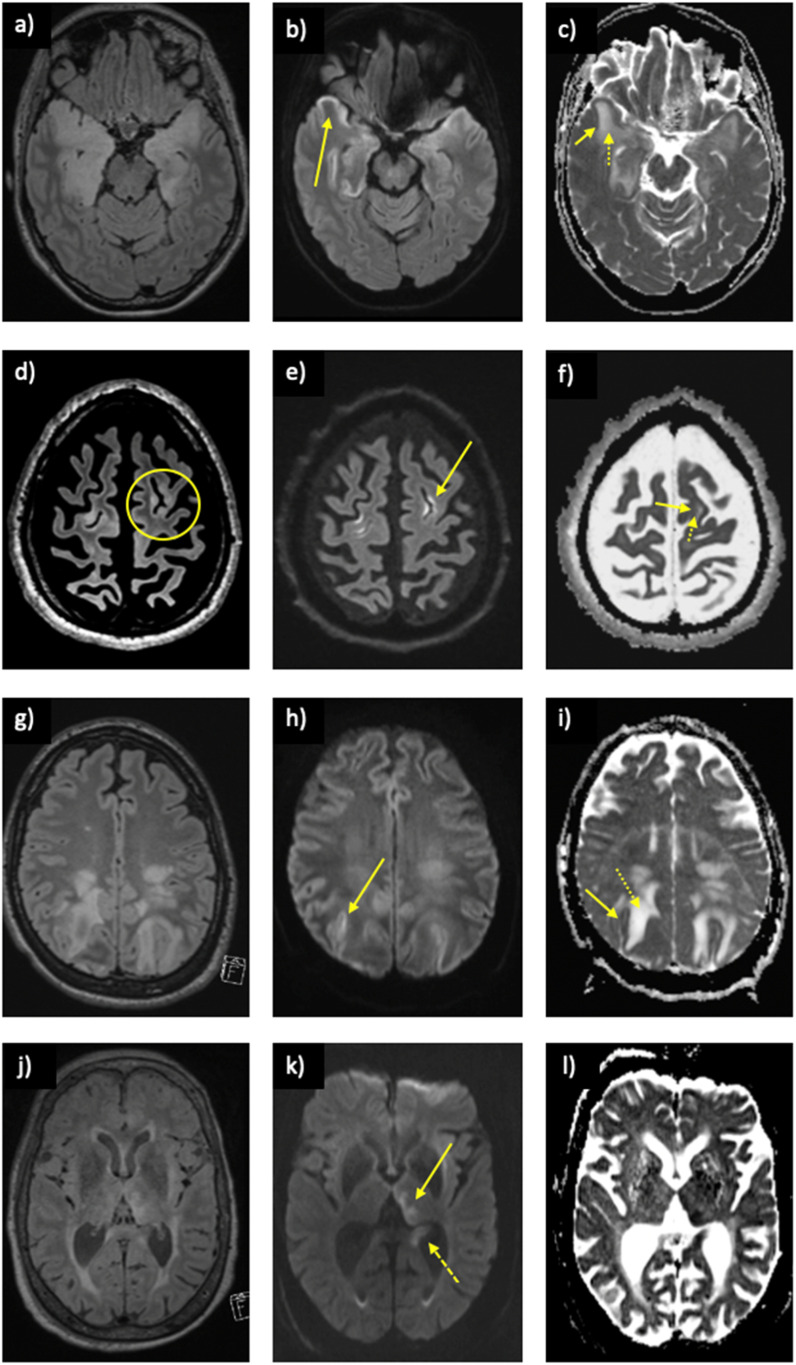
Other causes of encephalitis also demonstrating the split ADC sign. Herpes simplex encephalitis on (a) FLAIR demonstrating signal change within the temporal lobes bilaterally, (b) restricted diffusion on DWI in the cortex (arrow), and (c) the split ADC sign with low cortical ADC (solid arrow) and high subcortical ADC (dotted arrow). MELAS on (d) FLAIR demonstrating signal change within the cortex and subcortical white matter in the left frontal lobe (circle), (e) restricted diffusion on DWI in the cortex (arrow), and (f) the split ADC sign with low cortical ADC (solid arrow) and high subcortical ADC (dotted arrow). PRES on (g) FLAIR demonstrating signal change within the parietal lobes bilaterally, (h) restricted diffusion on DWI in the cortex (arrow), and (i) the split ADC sign with low cortical ADC (solid arrow) and high subcortical ADC (dotted arrow). Peri-ictal setting with a positive split ADC sign with concurrent evidence of a recent seizure including (j) FLAIR demonstrating signal change within the left pulvinar and hippocampus, (k) restricted diffusion on DWI in the pulvinar (solid arrow), and hippocampus (dashed arrow) with corresponding low ADC on (i).

In a different subject with seronegative autoimmune encephalitis, biopsy of the right frontal cortex revealed increased expression of glial fibrillary acidic protein (GFAP) signifying astroglial activation and gliosis. Lymphocytes were identified in the perivascular space, the vessel wall, and the brain parenchyma without specific findings to suggest vasculitis. Microglial activation without nodules was demonstrated. CD4+, CD8+, and CD20+ lymphocytes were identified. Axonal myelin loss was demonstrated within areas of necrosis.

## Discussion

In this study, we propose a novel visual MRI biomarker, the split ADC sign, and highlight its potential usefulness to suggest either acute encephalitis onset or to raise the possibility of an autoimmune etiology when location-based criteria are applied. When positive, the sign was present on the initial MRI performed upon patient presentation and can therefore be used to help focus further clinical and laboratory workup in the acute setting. The split ADC sign was found in five different entities causing clinical encephalitis and included infectious, metabolic, excitotoxic, and inflammatory etiologies (Figure 3). A split ADC sign was suggestive of autoimmune encephalitis when it was found to be scattered or multifocal throughout multiple brain lobes, with preferential involvement of the anterior cingulate gyrus, and in the absence of isolated temporoinsular (herpes simplex encephalitis) or parieto-occipital involvement (PRES). Furthermore, simultaneous pulvinar and hippocampal involvement was more suggestive of an isolated peri-ictal etiology.

All included cases of infectious encephalitis with a positive split ADC sign were attributed to the herpes simplex virus, although other viruses capable of producing this phenotype have been documented in the literature.^9^ The restricted diffusion of the cortex is likely due to direct viral invasion of the neuron, resulting in hijack of host cellular machinery, cellular energy failure, and cessation of normal cellular function.^10^ Cytotoxic edema ensues, beginning in the acute phase of the viral infection, and is most pronounced during the late acute and early subacute phases. Further works cite high cellularity and infiltration of neutrophils^11^ and lymphocytes^12^ contributing to the restricted diffusion seen in the cortex. Concurrently, the congestion, perivascular cuffing, and thrombus formation seen in the early acute phase of infection^13^ gradually decrease, resulting in increased perfusion and subsequent vasogenic edema. The edema is associated with increased ADC which predominantly affects the white matter.^14^ Towards the later phases (late subacute and chronic) of infection, the cytotoxic edema in the cortex transitions to vasogenic edema, leading to increased ADC and a gradual loss of the split ADC sign. This suggests a window in which the sign is positive in patients with herpes simplex encephalitis, determined by disease onset and the interval to MRI imaging. Furthermore, factors such as viral load, inflammatory response, general health, and age all contribute to the diverse disease phenotypes^12^ which may or may not manifest as a positive split ADC sign.

The split ADC sign was positive in patients with a recent seizure, that is, in the peri-ictal setting. As with herpes simplex encephalitis, pathomechanisms resulting in peri-ictal changes to brain diffusivity are a subject of debate. Seizures result in abnormal synchronous or markedly increased neuronal activity^15^ resulting in heightened energy consumption. This creates a relative mismatch between consumption and delivery of metabolites^11^ triggering reactive hyperperfusion to the brain region.^16^ Eventually, the Na+/K+-ATPase pump fails, resulting in an influx of sodium ions and water into the neuron, causing cytotoxic edema. The cortex, with higher energy demands than the underlying white matter, shows preferential involvement,^17^ while the white matter exhibits vasogenic edema, producing the split ADC sign. Similar to viral encephalitis, there appears to be a window in which the split ADC sign is positive in the peri-ictal setting. DWI restriction gradually resolves at day 14 or later, while T2W/FLAIR hyperintense signal change persists.^18^

In the peri-ictal setting, restricted diffusion is less pronounced, less common, and shows reversibility when compared to vaso-occusive ischemia.^15,18–20^ A proposed secondary mechanism involves excessive release of the excitatory neurotransmitter glutamate during seizures,^21^ leading to an influx of calcium ions and water, causing cytotoxic edema and restricted diffusion in a cascade collectively termed excitotoxicity.^22^ Further structures including the hippocampus,^23^ splenium of the corpus callosum,^22,24^ and thalamus^25^ can also demonstrate restricted diffusion in the peri-ictal phase. However, not all seizure activity results in restricted diffusion, and factors such as seizure duration (status epilepticus vs serial vs single seizure) and underlying brain lesions appear to play a role.^15^ Moreover, in the immediate peri-ictal phase, the subcortical region may appear hypointense on T2W/FLAIR due to increased oxygen extraction and predominance of paramagnetic signal change.^26^ In the end, it is impossible to predict which patients will develop the split ADC sign due to the complex evolution of ADC related to energy demand, supply, seizure severity, and seizure duration.^27^

In contrast, the cortical restricted diffusion in patients with posterior reversible encephalopathy syndrome (PRES) is etiologically different from viral encephalitis and peri-ictal restriction. PRES, a condition resulting in visual disturbances, headaches, vomiting, and seizures,^28^ is typically characterized by vasogenic edema, preferentially affecting the parieto-occipital lobes,^29^ although other regions such as the frontal lobe can also be involved.^30^ The cortical restricted diffusion in PRES occurs due to the vasogenic edema compressing the microcirculation resulting in cytotoxicity.^31^ Despite cytotoxic edema in the acute phase, PRES rarely progresses to infarction, in keeping with its known reversible nature.^32^ Microvascular compression-induced ischemia can be exacerbated by sympathetic-driven reactive vasoconstriction. However, the reduced sympathetic innervation and corresponding reduced autoregulation of the posterior circulation during hypertension are proposed as the main causes of PRES in the first place.^33–35^ Notably, the split ADC sign in PRES is likely underrepresented due to pseudonormalization of ADC, where cytotoxic edema cancels out on ADC through intravoxel averaging of vasogenic edema.^36^ Pseudonormalized ADC is associated with worse outcomes in PRES.^36^ Furthermore, the subcortical white matter can demonstrate low signal on DWI due to strong “T2 washout” effects.^37^

The cortical restricted diffusion in MELAS (mitochondrial encephalomyopathy, lactic acidosis, and stroke-like episodes) differs pathologically from viral encephalitis, peri-ictal restriction, and PRES. MELAS is an inherited mitochondrial disorder characterized by stroke-like episodes without associated vascular thrombo-occlusion.^38^ The acute phase demonstrates cortical restricted diffusion that does not adhere to vascular territories and is likely due to prolonged mitochondrial energy failure, resulting in Na+/K+-ATPase pump failure and cytotoxic edema.^39–41^ This predominantly affects gray matter due to increased energy demands and usually does not extend into the underlying white matter.^42^ Low ADC in the cortical ribbon gradually increases as hyperperfusion and associated vasogenic edema progress, resulting in pseudonormalized ADC. The relatively increased ADC in the early phase has been used to differentiate MELAS lesions from true ischemic lesions.^43,44^

Autoimmune encephalitis presents yet another unique etiology for the split ADC sign, likely encompassing elements from infectious, metabolic, and hemodynamic causes. Traditionally, cortical restricted diffusion was not associated with autoimmune encephalitis; in fact, its presence argued against autoimmune encephalitis when the temporal lobe was involved, favoring either herpes simplex encephalitis^45^ or seizure-related activity.^46^ However, cortical-restricted diffusion is increasingly recognized in autoimmune encephalitis through multiple case reports including anti-N-Methyl-D-aspartate (NMDA) receptor encephalitis,^47^ anti-voltage gated calcium channel (VGCC) encephalitis,^48^ anti-leucine-rich glioma-inactivated 1 (LGI1) encephalitis,^49^ anti-alpha-amino-3-hydroxy-5-methyl-4-isoxazolepropionic acid (AMPA) receptor encephalitis,^50^ and anti-glutamic acid decarboxylase 65 (GAD65) encephalitis.^51^ While hyperperfusion to the involved areas has been described,^51^ not all forms of autoimmune encephalitis demonstrate the split ADC sign.

At the cellular level, the diverse nature of antibodies in autoimmune encephalitis results in a range of effects on target antigens. Antibodies like anti-VGKC bind to the potassium channel and activate complement, resulting in neuronal destruction.^52^ Other antibodies, such as anti-NMDAR, decrease in the number of NMDA receptors on the synaptic surface, causing neuronal malfunction without complement activation or neuronal degeneration. These variations are further compounded by the differing mechanisms of blood brain barrier (BBB) breach by individual antibody subtypes, further contributing to the heterogeneous phenotypes in autoimmune encephalitis. While VGKC and NMDA antibodies infiltrate the BBB directly, GAD antibodies stimulate cytotoxic T cell infiltration of the BBB.^52^ Five of six patients in our cohort diagnosed with autoimmune encephalitis presented with seizures (two in status epilepticus) and a positive split ADC sign. Biopsy of two subjects revealed lymphocytic infiltration, suggesting that the split ADC sign likely arises from a combination of these factors.

Ultimately, this work is limited due to its single-center nature, and small cohort and sub-cohort numbers, which limits generalizability but is expected with rare diseases such as autoimmune encephalitis. For the same reason, we were not able to assess all known forms of antineuronal autoimmune encephalitis for the split ADC sign, and some entities are over-represented in our study (anti-GABA_A_R). Thus, larger cohort studies are required to validate the sign and differentiate between individual antibody syndromes. Moreover, we did not encounter any positive split ADC cases in a number of entities known to cause cortical restricted diffusion (such as acute disseminated encephalomyelitis (ADEM), Creutzfeldt–Jakob disease, hypoglycemia, and hyperammonemia); however, this does not rule out the possibility of a positive sign in these entities. While the retrospective nature of our study is advantageous for assessing rare diseases such as autoimmune encephalitis, it ultimately represents a limitation with regards to predictive performance of the split ADC sign.

To conclude, the goal of this work was to introduce a potential imaging biomarker in encephalopathy which can be used to suggest either acute encephalitis onset or an autoimmune etiology when location-based criteria are applied. The split ADC sign was positive in five different entities causing clinical encephalitis, including infectious, metabolic, excitotoxic, and inflammatory etiologies. A split ADC sign was suggestive of autoimmune encephalitis when it was found to be scattered or multifocal throughout multiple brain lobes, with preferential involvement of the anterior cingulate gyrus, and in the absence of isolated temporoinsular (herpes simplex encephalitis) or parieto-occipital involvement (PRES). Ultimately, prospective studies are required to validate the predictive performance and clinical utility of the “split ADC sign” in the acute setting.

## Appendix

### Abbreviations

ADC: Apparent diffusion coefficient
anti-GABA_A_R: Anti-γ-aminobutyric acid receptor type A
DWI: Diffusion weighted imaging
FLAIR: Fluid attenuated inversion recovery
MELAS: Mitochondrial encephalomyopathy, lactic acidosis, and stroke-like episodes
MPRAGE: Magnetization prepared rapid acquisition with gradient echoes
SWI: Susceptibility weighted imaging
